# Supplementary figures and images for: Behavioral and brain- transcriptomic synchronization between the two opponents of a fighting pair of the fish Betta splendens
Source: PLoS Genet. 2020 Jun 17;16(6):e1008831. doi: 10.1371/journal.pgen.1008831 (PMC7299326; doi:10.1371/journal.pgen.1008831)

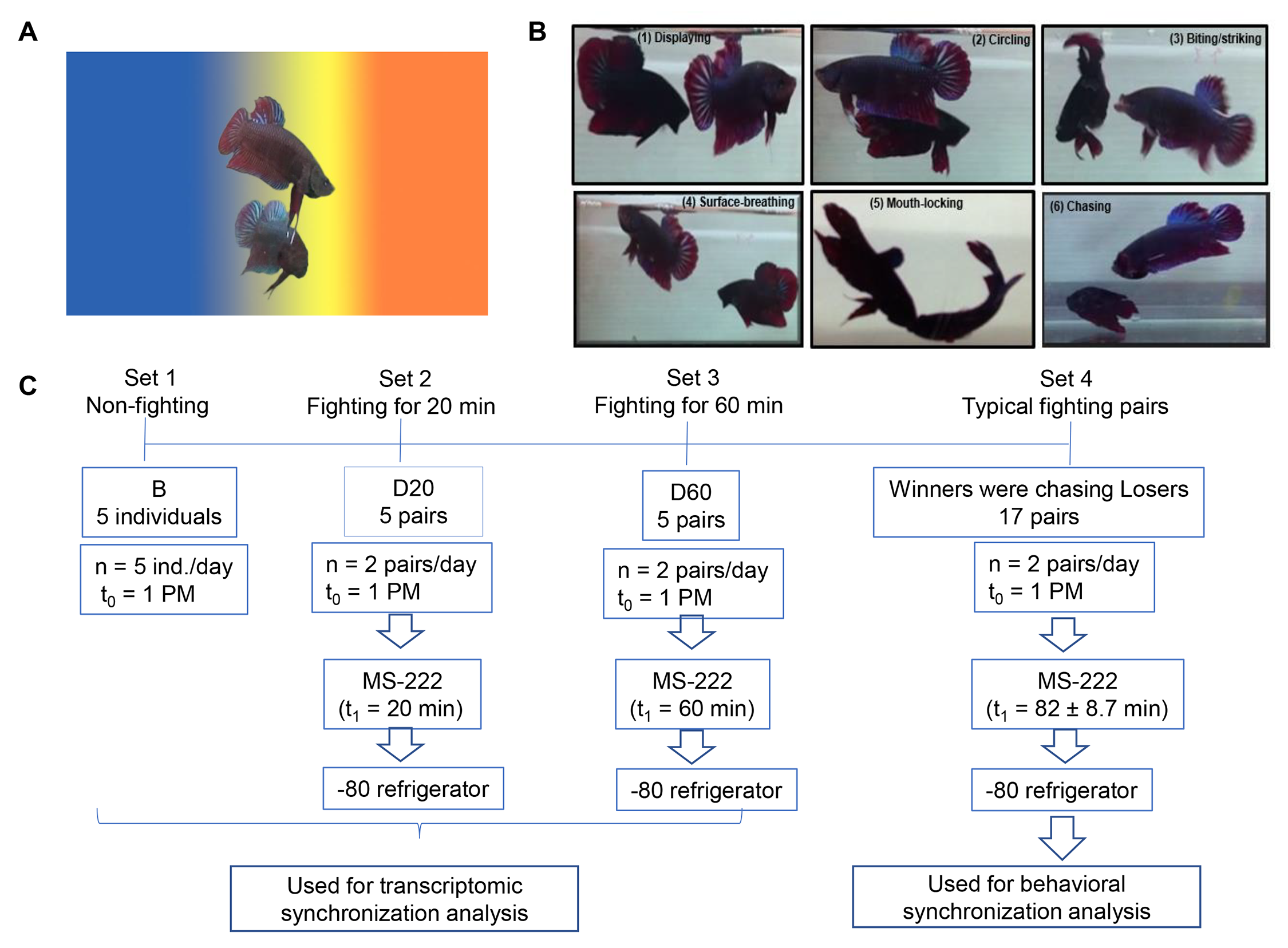

Supplement: S1 Fig — (A), Two male B. splendens. (B), Sequence of fighting behaviors that take place in order from (1) to (6) throughout the fighting process. (C), Sample collection. We conducted two fighting experiments (n) per day beginning at 1 PM (t0), and fish were immediately sacrificed at specific time points (t1) by submersion in the lethal dose of MS 222. It took 1 day to collect the five individuals for Set 1, 3 days to collect the five pairs for Set 2, followed by another 3 days to collect the five pairs for Set 3 and another 3 days for Set 4. After sacrifice, the samples were immediately transferred to a -80°C freezer and were stored there until subsequent brain dissection, RNA extraction and RNA sequencing. (TIF) [file pgen.1008831.s002.tif]

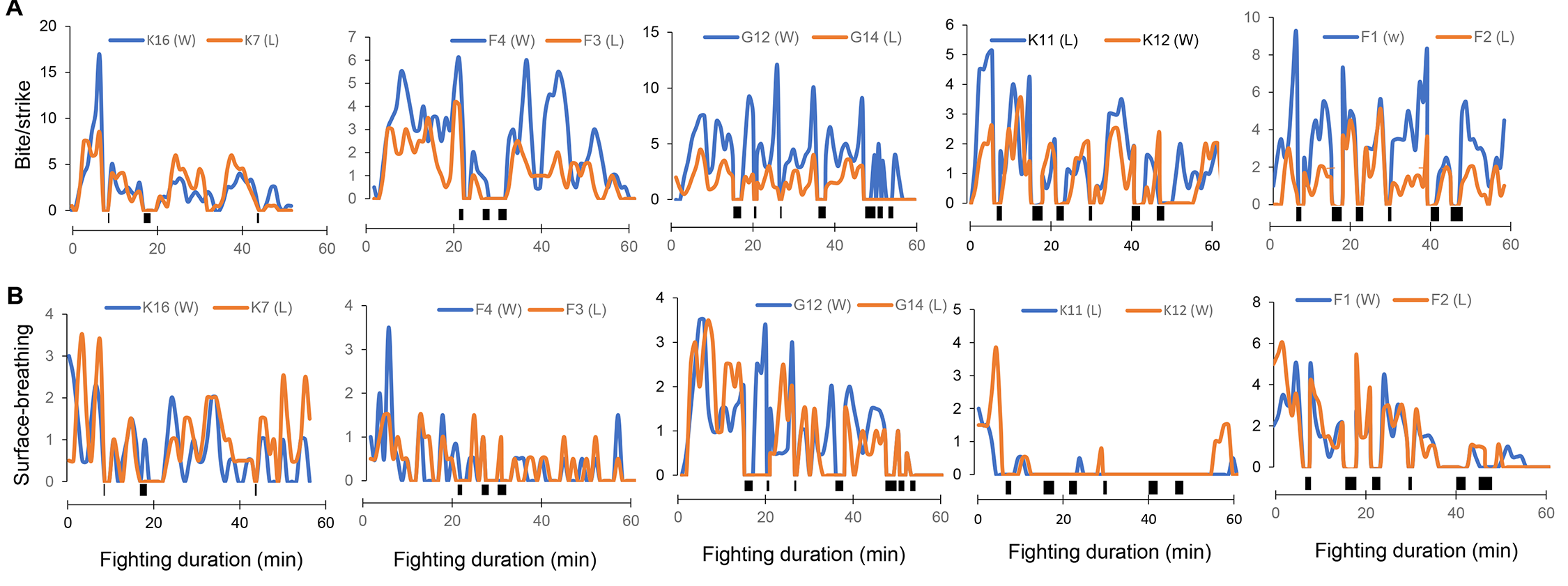

Supplement: S2 Fig — (A-C, G, H) Behavioral differences among all fighting pairs with respect to biting/striking. (D-F, I, J) Behavioral differences among all fighting pairs with respect to surface-breathing. These are data from five typical fighting pairs (Set 4 in S1C Fig) that were not included in the D20 or D60 group. (TIF) [file pgen.1008831.s003.tif]

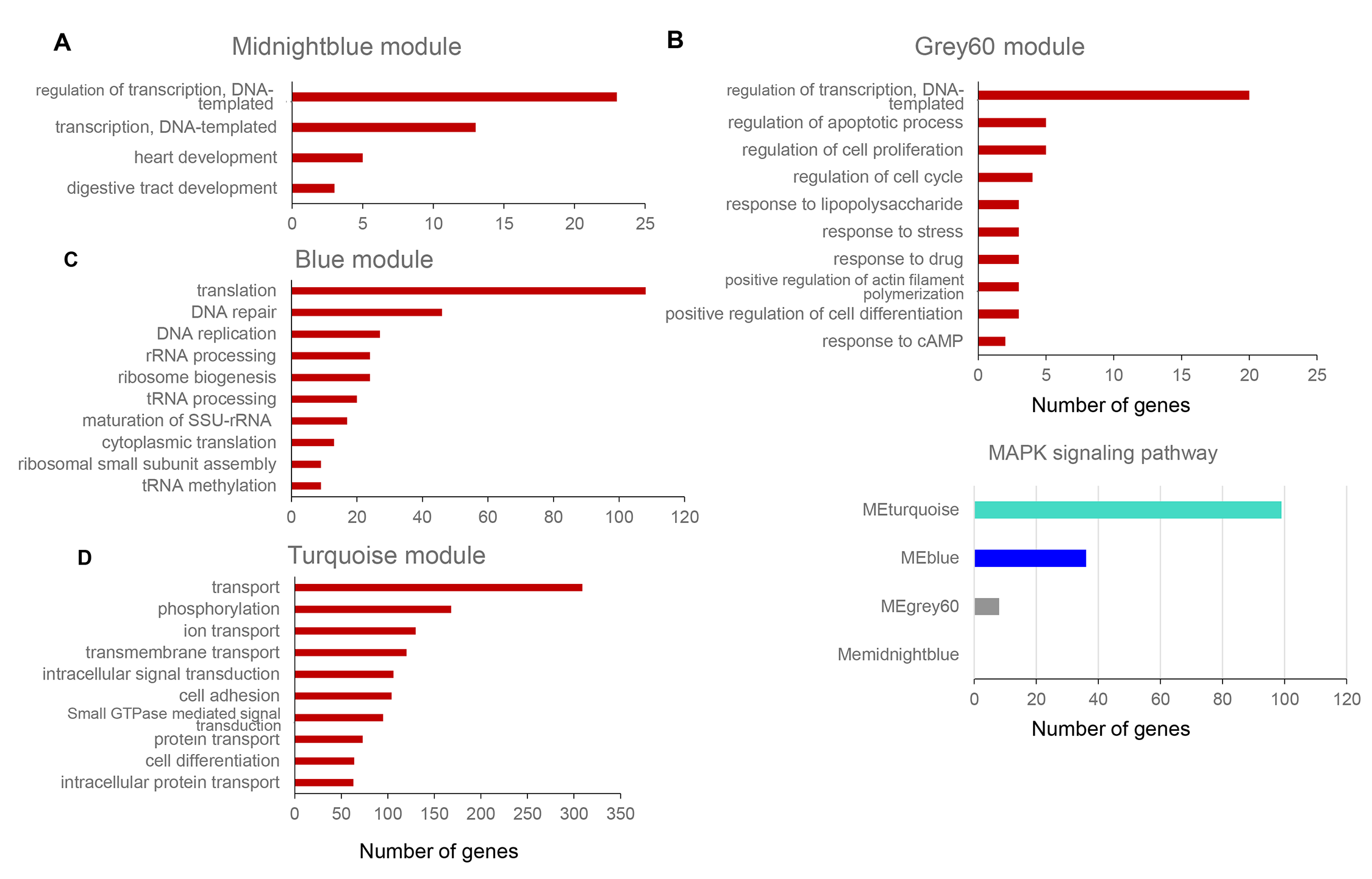

Supplement: S3 Fig — (A-E) Bar charts showing the enriched biological processes associated with four gene modules generated from the WGCNA analysis—MEmidnightblue (A), MEgrey60 (B), MEblue (C) and MEturquoise (D)—and the MAPK signaling pathway components associated with three of these modules (E). (F-I) Significantly enriched KEGG pathways for those modules associated with the fighting groups: MEgrey60 (G), MEblue (H) and MEturquoise (I) for the D60 and MEmidnightblue (F) for the D20; up-regulated genes are shown in red. (TIF) [file pgen.1008831.s004.tif]

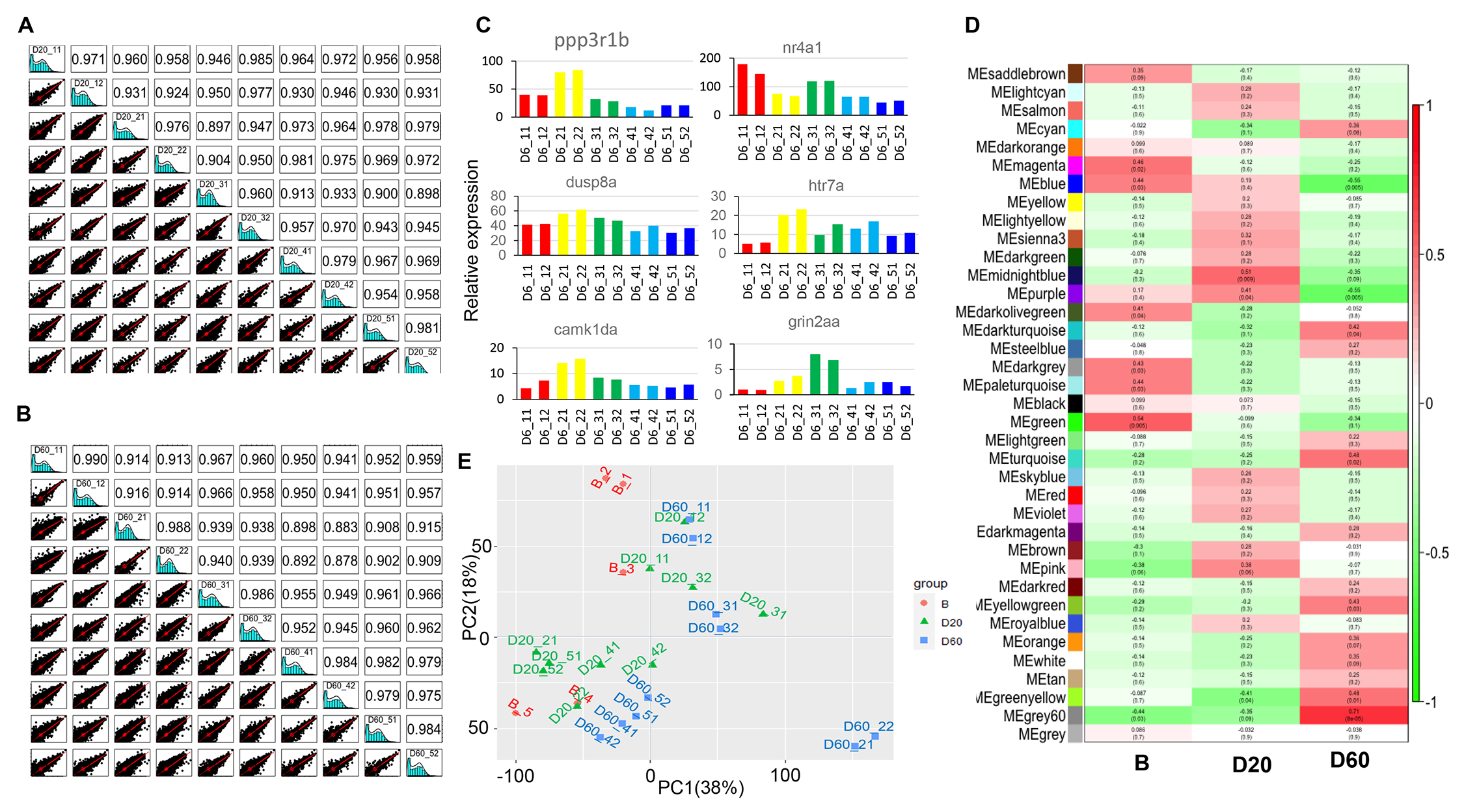

Supplement: S4 Fig — (A) The r values between the TMMs for the 23,306 gene transcripts from the two opponents of the fighting pairs in the D60 group; (B) The r values between the TMMs for the 23,306 gene transcripts from the two opponents of the fighting pairs in the D20 group. The r values in red boxes are for the fighting pairs. Bivariate scatter plots are shown below the diagonal, histograms are shown on the diagonal, and the Pearson correlation values are shown above the diagonal. (C) Representative genes showing pair-specific synchronization of expression. Although these genes were synchronized in all five pairs, the level of synchrony differed for each particular pair. (D) The WGCNA heatmap showing values for r (upper value) and p (lower value in parentheses) in all of the gene modules. (E) Clustering of all 25 brain samples using a principal component analysis (PCA); blue, non-fighting group (B); red, D20 group; and green, D60 group. (TIF) [file pgen.1008831.s005.tif]

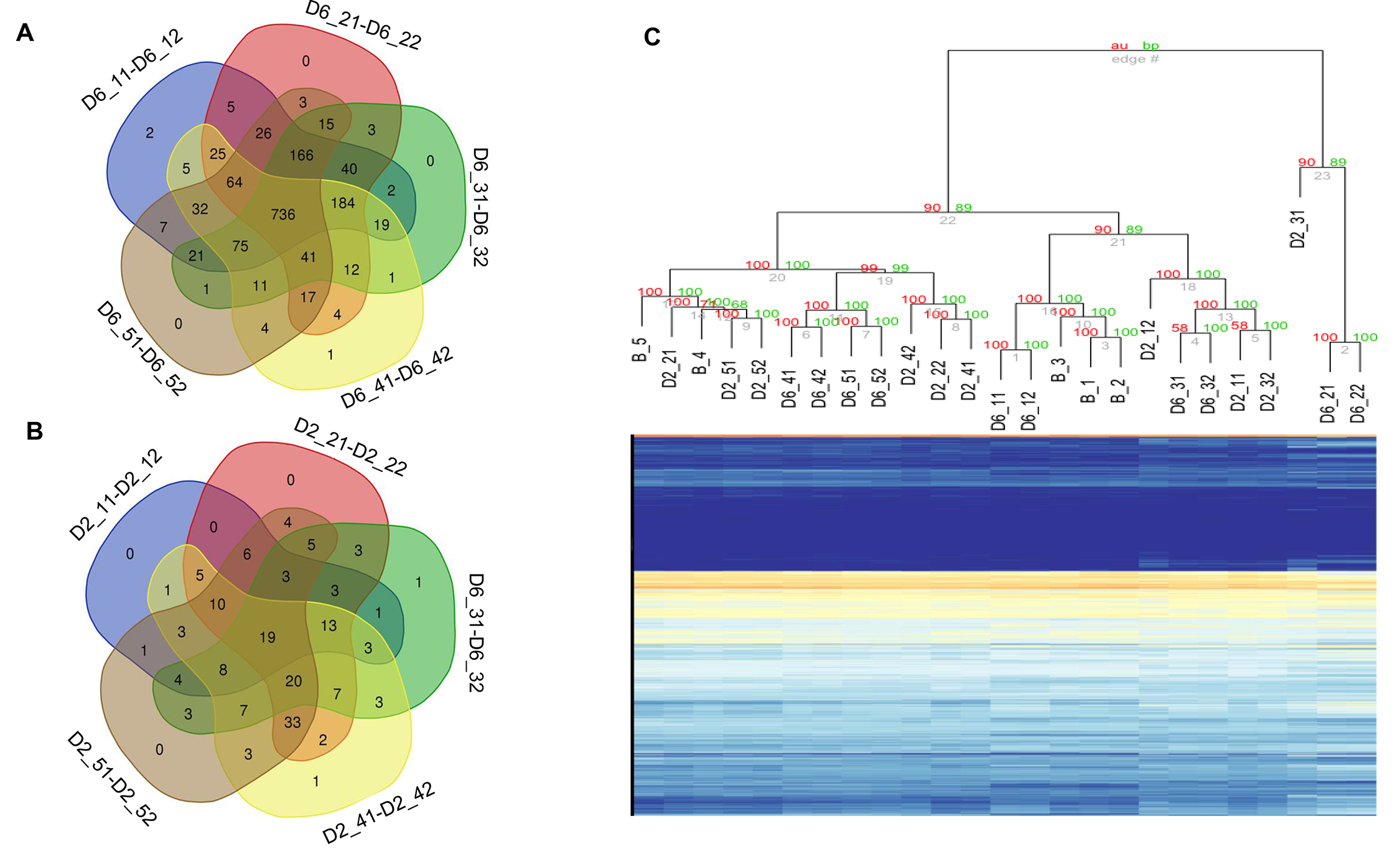

Supplement: S5 Fig — (A) Venn diagram generated from the 1,522 synchronized genes from five pairs of the D60 group (D60_11 vs. D60_12, D60_21 vs. D60_22, D60_31 vs. D60_32, D60_41 vs. D60_42, and D60_51 vs. D60_52); (B) Venn diagram generated from the 172 synchronized genes from five pairs of the D20 group (D20_11 vs. D20_12, D20_21 vs. D20_22, D20_31 vs. D20_32, D20_41 vs. D20_42, and D20_51 vs. D20_52). (C) Heatmap using the 25 cDNA libraries with each of the 23,306 gene contigs (all gene set). Intensity of color indicates expression level (red, high expression; blue, low expression). Similarities between individuals within fighting pairs and between fighting pairs as shown by hierarchical clustering can be seen above the heatmap. Bootstrap values at the nodes were obtained by hclust function in R. (TIF) [file pgen.1008831.s006.tif]

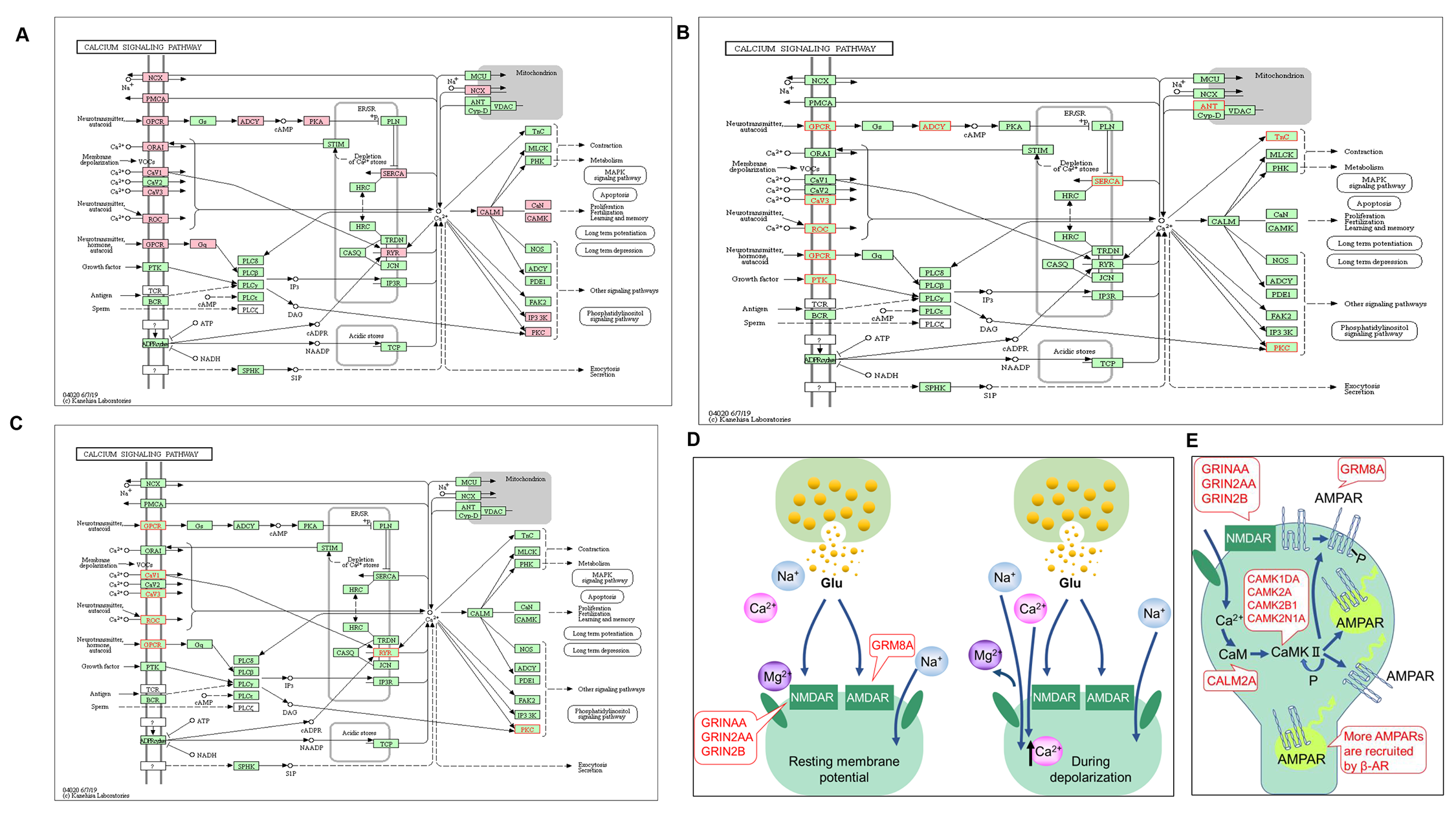

Supplement: S6 Fig — (A-C) Calcium pathways generated from the synchronized gene set in the D60 group and non-synchronized gene sets of the D20 and D60 group (B, C). (D) Glutamate (Glu) is released from the presynaptic terminal and acts on both postsynaptic N-methyl-d-aspartate receptors (NMDARs; grinaa, grin2aa, grin2b subunits) and α-amino-3-hydroxy-5-methyl-4-isoxazolepropionic acid receptors (AMPARs; grm8a subunit) to depolarize these receptors and release Mg2+. (E) Ca2+ increases in the postsynaptic terminal and binds to Calmodulin (CaM; calm2a subunit) to activate the calmodulin-dependent protein kinase (CAMKII; camk1da, camk2a, camk2b1 subunits). Also, β-adrenergic receptors (β-ARs) are used to recruit more AMPAR to promote long term memory. (TIF) [file pgen.1008831.s007.tif]

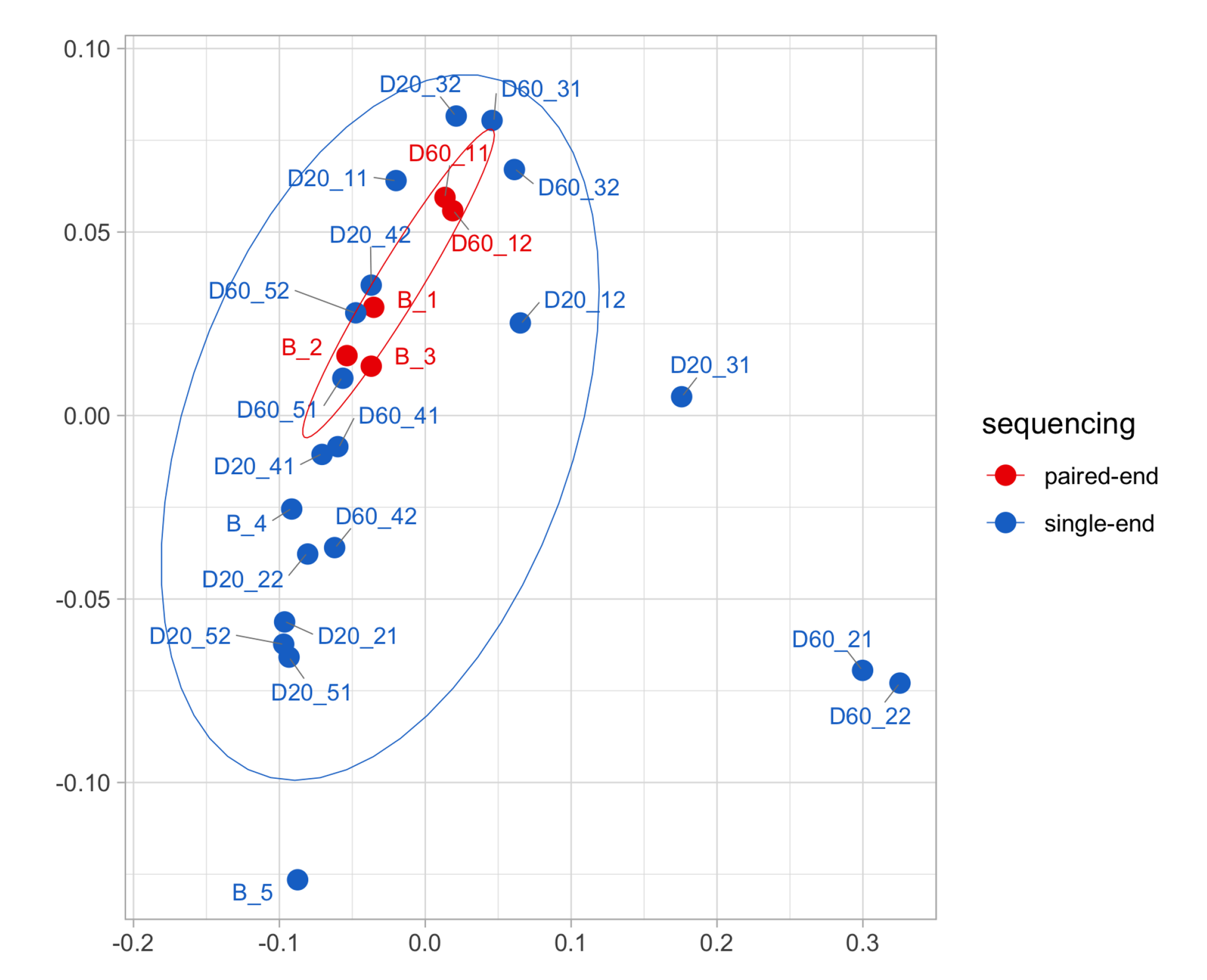

Supplement: S7 Fig — B, non-fighting group; D20, the D20 fighting group; D60, the D60 fighting group. Red circle clusters the paired-end sequencing and blue circle clusters the singe-end sequencing. (TIF) [file pgen.1008831.s008.tif]
