# Supplementary material for: Behavioral and brain- transcriptomic synchronization between the two opponents of a fighting pair of the fish Betta splendens
Source: PLoS Genet. 2020 Jun 17;16(6):e1008831. doi: 10.1371/journal.pgen.1008831 (PMC7299326; doi:10.1371/journal.pgen.1008831)
Supplement: Attachment — Submitted filename: response_to_reviewers.docx [file pgen.1008831.s024.docx]

**Responses to the comments of the reviewers**

**Dear** **Drs. Alison Bell (Guest Editor) and Gregory Barsh (Editor-in-Chief):**

We greatly appreciate the critical but constructive comments made by you and the three anonymous reviewers, which were very helpful in improving our manuscript. We have tried our best to respond to these comments and have revised our manuscript accordingly. Of course, we would appreciate further comments.

Before we present our point-by-point responses to the reviewers’ comments, we focus here on three main points summarized by the Guest Editor.

**First of all, we attempted to explain why we think that brain gene expression is operating over a time scale relevant to behavioral synchrony during a fight and why brain gene expression (as opposed to, say, neural activation) was measured.** We are interested in elucidating what happens in the brain transcriptome of two fighting opponents of the fish *Betta splendens* during fighting. As molecular biologists, we obviously focus on gene expression, whereas neural activation is beyond the scope of our specialty. We first observed that gene expression data for the two opponents within a fighting pair were always clustered together based on a heatmap clustering analysis. Also, our data from the behavioral analysis suggested that *B. splendens* express different sets of behaviors during fighting and that the mouth-locking behavior has an important role as a constraint on these behaviors.

In addition, we observed that at the very beginning of the fight *B. splendens* mainly display their fins, during fighting they chiefly perform bite/strike/mouth-lock/chase, and after fighting they show chase/flee/retreat. Thus, at first it seemed quite simple for us to hypothesize that each fighting stage might have unique gene expression data associated with it. Additionally, there are transient periods between fighting stages during the fight, and some of the demands of fighting such as biting, striking, chasing, surface-breathing, etc., remain constant across these stages. Thus, one might expect to see the brain signal of a preceding fighting stage persist into subsequent stages, resulting in shared gene expression among fighting stages in the series, with the expression of these genes being either transient between temporally close stages in the series or persistent across all fighting stages.

We subsequently learned that previous studies that measured neural activation provided strong evidence for the synchronization of neural activity across the brains of two interacting mice or bats, showing that there is a positive correlation between the neuron activity correlation and behavior correlation between two interacting individuals (refs. 38 and 39) (the reference numbers refer to references listed in the main text of the manuscript). In addition, it has been reported that different neuronal activity patterns induce different sets of activity-regulated genes (ref. 94). Several lines of such evidence support the idea that a series of neuronal activity patterns can be inferred from the patterns of gene expression.

Collectively, these prompted us to hypothesize that brain gene expression may be relevant to behavioral synchrony during a fight in *B. splendens* over that time scale.

**Second, we address the issue of there being “no follow-up studies presented, nor attempts at validation”.** We do believe that our finding about the strong correlations between behavioral synchronization and transcriptomic synchronization in a pair-specific manner should be validated per se. Here, we do not make any judgment about the causal relationship between these forms of synchronization. Actually, we do not know whether the genes that were synchronized in the brains of the fighting pairs were causally related to behavioral synchronization or if they were synchronized as a consequence of behavioral synchronization. Although it might be possible that manipulative experiments that change the expression of particular genes can help disentangle causes from consequences in some cases, it is becoming increasingly evident that perturbations to a single candidate gene for complex traits such as aggression might not be fruitful, because the behavior reflects the coordinated action of an entire network of genes. Thus, although we agree that gene expression profiling studies should have follow-up experiments to provide insights into biological/genetic mechanisms, we admit that this is beyond the scope of this study given the complexity of the thousands of genes that contribute to brain-transcriptomic synchronization. We think that not only our study but also other studies such as the study in mice (ref 71) could also encounter the same challenge in terms of gene validation. We are now working toward finding solutions to overcome the challenge by measuring neural activation using markers of neural activity (e.g., *egr1*, *c-fos*) with the in situ hybridization technique as the first step.

**Finally, we present a justification for performing whole-brain analysis and explicitly state which brain regions might be good candidates for future study.** In the revised version of the manuscript, we made a justification for the whole-brain analysis. We noted that none of the well-known candidate genes for aggressive behavior in zebrafish were identified in our comparison, such as *tphs*, which has a role in aggression (ref. 28). It is possible that several aggression-associated genes were up- or down-regulated in a specific part of the brain in our fighting system but could not be detected in our study of whole-brain gene expression possibly because the effect was diluted, showing the limitation of using the whole brain. We also mentioned that studies focusing on the specialized parts of the brain known as the “social behavior network”, a collection of brain nuclei that regulate social behaviors across vertebrates, have been more fruitful (refs 70, 80-82). Furthermore, we proposed that the telencephalon region could be a good candidate for further analysis because it contains structures that are homologous to the hippocampus and the amygdala in mammals, brain regions associated with aggression in other vertebrates. Particularly, several crucial loci in this region such as the dorsal medial (associated with the fear response), the dorsal lateral (associated with spatial learning), lateral spectrum (associated with anxiety), the pre-optic area (associated with reproduction), the cerebellum (associated with attention) should be analyzed more closely (please see the Discussion).

One of the improvements in this revised version is that we re-designed the statistical test for brain-transcriptomic synchronization. Specifically, to assess how similar the transcriptomes of paired fish are, we have revised the analysis to compare the paired fish to unpaired fish from the same treatment (e.g. D20-paired vs. D20-unpaired and D60-paired vs. D60-unpaired) using the permutation test instead of the t-test that was used in the previous manuscript. We also omitted the comparison with the non-fighting fish that we had included in the previous manuscript. Furthermore, we used the permutation test to set the threshold for identifying synchronized genes and described these thresholds in detail in the Methods. We have re-analyzed our data for the identification of synchronized genes, re-made Fig 6 by adding some representative genes showing the pair-specific individualization brain-transcriptomic synchronization, and simplified the schematic of the calcium pathway. We then reviewed our interpretations of the findings in this revision accordingly.

Moreover, we re-designed the linear discriminant function analysis (LDA) and re-made the LDA figure using the top 50% most variable gene transcripts (11,653 genes) instead of using the top 10,000 highly expressed genes (Fig 2B). In Fig 2, we also added a volcano plot showing differentially expressed genes (DEGs) from the comparison of B vs. D20 and a plot in which the D20 DEGs are projected on to the D60 DEGs and vice versa. Another improvement is that we added bootstrap values to Fig 3C and Fig 5B. The Fig 5B was re-made using the top 10% most variable gene transcripts (2,330 genes) to exclude those that showed minimal variability and had been included in the previous manuscript.

Last, but not least, we added more detailed information in the Methods and provided source code files to assist other researchers with understanding and replicating our experiments. We changed the term “neurotranscriptomic” to “brain-transcriptomic” given that brain samples include transcripts from many non-neuronal cells that also have dynamic responses to stimuli and may play important functional roles. We removed the promoter region analysis as it did not provide important information, and we also removed mention of the “mirror neuron” from the Discussion, given that this concept is quite contentious among systems neuroscientists.

Below are our point-by-point responses to the comments:

**Reviewer #1:**

**As a reader, it is crucial for me to clearly see in a ms what we know on the topic and what we don’t, and why its important to know more about this. In the ms I did not see a clear reason for studying gene expression rather than neural activation. Telling the reader what the ms will bring is essential. I was also surprised that there were no clear hypothesis nor predictions. I have several questions about the methods.**

Specific comments

Introduction

**After line 100, it would be essential to explain why the authors want to learn about the neurotranscriptomic state of males during a fight. Why should we study this, what will it bring to our understanding of behavior in general? This is important for the broad audience that is targeted by this journal. It would be essential to have hypotheses and predictions, at least proposing alternative models of what should happen, and in which type of genes, given the behavioral data that is available and what we know from studies in males that have already resolved conflicts (dominant/subordinate for example).**

We appreciate this comment. In this revision, we have provided more information about our hypotheses as follows. Please also see the Introduction (Lines 105-114).

**Before revision:** No sentences to be mentioned.

**After revision:** Behavioral and physiological synchronization have long been studied by scientists given its importance in reproductive/mating behavior, cooperative behavior, aggressive behavior etc., (33, 34). Some crucial signaling molecules such as serotonin (5-HT) or Arginine-vasotocin (AVT) has been known to modulate the levels of such cooperation at physiological level (35-37). Furthermore, recent studies have provided excellent physiological evidence for such synchronization at the neuronal level (38, 39) provided excellent physiological evidence for such synchronization at neuronal level (38, 39). Collectively, decades of behavioral evidence as well as these recent physiological findings suggest that synchronization at the gene expression level might also occur.

**Line 106-108 and results line 150-153**

**For the whole ms****, it is not clear if conflicts were resolved or not in the D20 and D60 fish. If not, can an index of escalation or of when in the timeline the pairs are towards resolution (creating an index based on the “typical fighting pairs” maybe?) be created? This analysis looks like a time series analysis, but we are not sure if each pair characterised as “D20” and “D60” is actually in the same stage. Were they always killed while mouth locking? Etc?**

As you noted, we had not clearly stated whether conflicts were resolved in the D20 and D60 fish in the previous version of the manuscript. In this revision, we have added an explanation that the conflicts were not resolved in all pairs among the D20 and D60 fish (see Methods, line 542-549)), and we also provided an explanation as to why the D20 and D60 pairs were in the same stage. From our results shown in Fig 1C and 1D, we reasoned that the fighting pattern between two opponents between the two fish in each fighting pair looks the same, and that the fighting sequences were consistent between pairs throughout the 60-min fight duration. Thus, it is likely that each pair characterized as “D20” and “D60” could actually be in the same stage

The escalation index that you suggested could be generated following the procedure described in ref. 44 as follows:

**Escalation index** = overt aggression/(overt + ritualized aggression) = (bite + chase + strike)/[(bite + chase + strike) + (display + circle)]

To obtain this index, we would need to quantify several ritualized aggressive behaviors (display, circle, fin spread, and others), which would require additional efforts for the behavioral analysis and statistical test due to the resulting complexity.

In our study, instead of creating an escalation index as mentioned above, the resolution time was actually identified after detailed scrutiny of behavioral patterns exhibited during interactions of 17 fighting pairs (Table S1). We have determined that the resolution time in our fighting system occurred after 60 min of fighting.

**Results**

**Line 154**

**Please explain why the top 10 000 highly expressed genes were used. And what does highly expressed mean? Highest read count? What does it mean biologically?**

We have learned that our approach in the previous version of the manuscript was not appropriate, as you have pointed out. We made several changes in the Methods and also re-made Fig 2B. We chose the top 50% most variably expressed genes (11,653 genes, see Methods) instead of the top 10,000 most highly expressed genes.

**Before:** Linear discriminant function analysis (LDA) using the input variables of the top 10,000 highly expressed transcripts from the 25 libraries was used to differentiate the fighting groups (see Methods)

**After:** Linear discriminant function analysis (LDA) using the top 50% most variables of the gene transcripts from the 25 libraries was used to differentiate the fighting groups (see Methods, **line 160-162**)

**Line 178-179**

**Why discuss the fact that some genes have a particular biological function? Is this the result of an enrichment analysis or just cherry picking genes?**

**In the revised version of the manuscript, we emphasized** that some of these genes have a particular biological function given that they were previously shown to be involved in these functions, and we have added references to these statements. Those particular genes were chosen for attributes of their expression across the fighting stages and known relevant functions in previous studies.

**Before:** Did not mention the reason why we discussed about these genes

**After:** In addition to the attributes of their expression across fighting stages, several of these genes are known to associate with neuronal activity (e.g., *c-fos*, *fosb*, *egr4*), neural plasticity processes related to learning and memory (e.g., *bdnf*, *npas4*, *nr4a1*), neurotransmitter (e.g., *grin2a*, *grik2*) and epigenetic function (*rnf44*) (Fig 3B and see Discussion) (6, 48) (refs 6, 48) (Line **187-190**)

**Line 181**

**How can duplicates exist? What do they represent? Would it be better to remove them before the statistical analysis of DEG?**

Yes, we agree that duplicates should have been removed before carrying out the DEG analysis. Thus, we removed the duplicates and repeated the analysis for this revision and re-made (Fig 3C).

**Before:** The heatmap generated from the total of 961 unique DEGs (after duplicates were deleted from the initial 1,082 DEGs) illustrated four main patterns of gene expression that were distinguishable among the fighting groups (B,183 D20, and D60) (Fig 3C)

**After:** The heatmap generated from the total of 1,082 DEGs illustrated four main patterns of gene expression that were distinguishable among the fighting groups (B,183 D20, and D60) (Fig 3C) (**Line 191, 192**)

**Line 195**

**The WGCNA is not explained in the methods and we have no idea how it answers the question about the neurotranscriptomic state of fighters.**

We learned that we mistakenly left out the WGCNA description in the Methods in the previous version of the manuscript. We have provided a detailed explanation about it in this revision and included the source code file for replication. Please refer to the Methods (**Line 657-670**).

**Line 200**

**Please explain how a gene module eigen value can be correlated with 3 treatments. How can one correlate continuous values with 3 discrete groups? What can of correlation can be used if one of the variables has no value / cannot be ordered?**

We believe this concern results from the missing information about the WGCNA in our previous manuscript. Please have a look at the Methods for the explanation about the WGCNA as noted above. We expect that this will alleviate your concerns, but if it does not please kindly let us know. (**Line 657-670**)

**Line 218-224**

**What is the meaning of these results? What question do they answer?**

We have clarified the ambiguity of this point in the revised manuscript by stating that these two methods use different approaches but reach the same conclusion, which strengthened our findings, as one can corroborate the other. Please see the Results section.

**Before:** Did not cover

**After:** Together, the DEG and WGCNA analyses used different approach but overall showed a high agreement in the characterization of the transcriptome changes associated with each fighting group across the fight. (**Line 219-221**).

**Line 317-337**

**This analysis really looks circular to me. The authors need to explain why extracting genes based on their function (learning and memory) are then used in a kegg analysis to find enriched pathways (?) and one of these pathways but not the others (why?) related to calcium signalling pathway is then used to analyse the gene sets (including the one from which the genes related to memory where extracted from in the first place!) to determine if each gene set is enriched for genes related to learning. Please explain how this is not circular.**

Yes, we agree that our original description was circular. We have revised the text in this version of the manuscript to avoid circular interpretation. Basically, after finding that several synchronized genes in the D60 group that were up-regulated encode proteins involved in the calcium pathway related to learning and memory, we subsequently used the same method to generate calcium signalings for the other three gene sets (D20 synchronized genes, D20 non-synchronized genes, and D60 non-synchronized genes) to examine if these gene sets involve in memory formation. Please refer to the Results. (**Line 305-314**)

**Before:** We examined what gene sets among the three groups (1,165 synchronized genes in D60, 213 synchronized genes in D20, and 1,575 non-synchronized genes in both D20 and D60) selected under the criteria of FDR < 0.05 and log FC > 0 were involved in the calcium signaling pathway using KEGG pathway analysis. Here, the D60 synchronized gene set were evaluated twice.

**After:** Next, we used the calcium pathway as a proxy for learning and memory examination for other gene sets. We examined what gene sets among the three groups (104 synchronized genes in the D20, and 567 non-synchronized genes in the D20 and 791 non-synchronized genes in the D60, the D60 synchronized genes from which the genes related to memory where extracted from in the first place was no longer examined) were involved in the route leading to learning and memory. Here, the D60 synchronized gene set was excluded.

**METHODS**

**Line 604**

**Why use a cut off of FC > 2?**

This was an error in the previous manuscript. The cutoff should have been |logFC| > 2, which we have changed in this revision.

**Before:** The DEGs were obtained from the comparison of gene expression between the non-fighting group with the D20, and the non-fighting group with the D60 using the criteria of FDR ≤ 0.05 and log FC > 2 (did not include the absolute value sign) by the edgeR package in R (89) (Supplementary Table S6).

**After:** The DEGs were obtained from the comparison of gene expression between the non-fighting group (B) with the D20, and the non-fighting group (B) with the D60 using the criteria of FDR ≤ 0.05 and |log FC| > 2 (include the absolute value sign) by the edgeR package in R (Supplementary Table S6, source code file 1). (**Line 630**)

**Line 606-607**

**If 45 pairwise correlations are done, one needs to correct for multiple testing?**

In this revised version of the manuscript, we used a permutation test instead of the t-test that was used in the previous version. Thus, the correction for multiple testing is now included.

**Line 610-611**

**Please give more detail for the LDA and GoM, presently they are impossible to replicate**

Thank you for your comment. We have given more detailed information for the LDA and GoM analyses in the Methods and have provided the code for replication in this revision. Please see the subheadings “clustering” (**Line 644**) and “WGCNA” (**Line 657**).

**Line 612-614**

**The heat map combined a k-mean clustering and hierarchical clustering according to the figure caption. Please give detail on the parameters used for the hierarchical clustering (distance measure, model for clusters) and for the k-means (how were the number of k clusters chosen?). Also, how where the TMM data normalised?**

Thank you for this suggestion. We have re-made the figure with the heatmap and have described the methods in more detail in the Methods section of this revised manuscript. The TMM normalized data are described in the “Quality Processing and Read Mapping” section in the Methods, and we have also provided source code for this analysis in this revision.

**Line 651**

**The linux pipeline should be on github or another repository for real reproducibility rather than available on request (File Sxxx)**

We have changed the text accordingly. The source code files are now available on Github (<https://github.com/Amber-Vu/Fighting-fish-1>). All other relevant data are available upon request. (**Line 718**).

**Figures**

**Figure 2B is of poor quality**

We have re-made this figure.

**Figure 3A the venn diagram is for all genes, no matter in which direction they changed expression? Are they always in the same direction in D20 and D60 compared to controls?**

The Venn diagram shown in Fig 3A is for all DEGs including both up- and down-regulated genes. Yes, these DEGs are almost always in the same direction in the D20 and D60 **groups** as compared with controls, which we now show in Fig 2E (please have a look).

Minor comment

**Line 332**

**Here it says log FC > 2, probably mean log FC > 0 ?**

Actually, when we mentioned that we had used more stringent criteria in the previous manuscript, we meant that instead of using a relaxed threshold for the DEG analysis as log FC > 0, we used log FC > 2. However, after we re-designed the threshold for identifying synchronized genes in this revision, we realized that this statement is no longer supported, so we have deleted that text entirely.

**Before:** Moreover, when the three gene sets mentioned here were analyzed using the more stringent criteria of FDR < 0.05 and log FC > 2, fewer highly expressed genes were found to be involved in the calcium signaling pathways.

**After:** Did not cover

**Line 582**

**The authors refer to sample D50_21 and D50_22 but I suppose they mean D60?**

Thank you for the comment. Yes, this was an error in the previous manuscript; we have changed the text accordingly.

**Reviewer #2:**

This manuscript analyzes behavior and gene expression changes in fighting fish. The manuscript is generally well-written and includes sophisticated analyses to address novel questions. My enthusiasm for the paper is reduced by several analysis issues that leave me wondering how many of the findings are trivial consequences of the analyses vs. findings of true biological significance.

General concerns about behavioral synchronization findings:

**(1)** **To what extent is the calculated synchronization a trivial consequence of the inherent synchrony of mouth locking + the reality that other behaviors occur in a defined order vs. a more active form of synchronization?** **Is there a way to evaluate the expected level of synchrony that considers mouth-locking as a constraint?**

Basically, our data showed that the first mouth-locking started at 10.63 ± 2.10 min, continued for 1.29 ± 0.24 min, and occurred on average 12.12 ± 2.4 times during the 60-min fighting period (Fig 1B, Fig S2). Thus, mouth-locking accounted for 26.88% of a 60-min fighting duration. As surface-breathing and biting and striking are restricted during mouth-locking, the frequency, duration, and distribution of mouth-locking behavior during the fighting period determine the fight structure and level of behavioral synchrony.

The fighting process does, however, involve several other behaviors, some of which might also be involved in coordination such as tail-beating, body-orientation, body-coloration, etc. Including these behaviors in the analysis would require more advanced tools given the increase in complexity. Thus, it is difficult for us to distinguish between a constrained and predictable set of behaviors (that thus induce similar changes at the level of gene expression) versus an active synchronization process. Also, it is difficult for us to evaluate in detail the expected level of synchrony that occurs when mouth-locking is a constraint. We think that we should take your suggestions into deep consideration during our future studies.

**(2) The methods mention a 1-minute overlap for analysis, but I was not sure what this meant. Please explain more fully the justification for the window approach.**

In this revision, we have explained more fully the justification for the window approach in the Methods as follows. To consider the entire fighting process, the time series was broken up into short, overlapping segments (or time windows). Calculations of the frequency and duration of behaviors were then carried out for each of these time windows (1 min in our case). The resultant time sequence of all frequency and duration values from each time window can indicate behavioral changes over time. We chose the length of the time windows based on the type of movement**s** being considered and the observed time scale for behavioral changes. This approach is well established for nonstationary statistics that vary over time and has the advantage that the investigator can control the time scale for behavioral parameter estimation by choosing an appropriate time window length (please see ref. 99 for more details) (**Line 569-574**).

**General concerns about DEG analyses:**

**(1) Given the experimental design, I would recommend an ANOVA rather than two pairwise tests. ANOVA would more appropriately take into account the use of the same control group for the D20 and D60 groups & would allow a formal assessment of differences between D20 and D60 directly rather than making inferences based on the lack of or presence of overlap in separate DE analyses. Also, I suggest those models take into account the non-independent nature of the paired fish (e.g. a random effect for pair).**

We think that your comments might refer to the Fig 3A in which you suggested us to carry out an ANOVA test with three groups (control, D20, and D60) rather than carrying out a pairwise test (a t-test) with two pairs (control vs. D20 and control vs. D60). If it is the case, we want to explain that we do not apply any statistical test in this regard. What we showed is that the DEGs of the B vs. D20 group and the B vs. D60 group were first obtained by the edgeR package in R (as described in the Method), then the numbers of shared genes between these two fighting groups (D20 and D60) was shown by overlapping these two gene lists of DEGs. Noticeably, we newly added that to examine whether the number of 295 overlapping DEGs between the D20 and D60 was greater than expected by chance we performed hypergeometric test, which was conducted by the *phyper* function in the “dplyr package” in R. We showed that these 295 genes were shared much greater than expected by chance (p < 7.2e-298). (**Line 185, 186**).

Could you please kindly let us know if our answer is exactly about what you were asking? or which particular text/figure in the ms we need to look into?

**(2) I read Figure 2A quite differently than the statements on lines 155 – 158. As I see it, Axis 1 separates D20 and control from D60, and Axis 2 separates D20 and D60 from controls.**

Thank you for your comment. This was an error in the previous manuscript, and we have corrected the text in this revision accordingly.

**Before:** This analysis revealed two significant functions: Function 1 (LD1), which separated the D60 group from the non-fighting group, explained 67.9% of the variance, and Function 2 (LD2), which separated the D20 group from the non-fighting group, explained 32.1% of the variance (Fig 2B)

**After:** This analysis revealed two significant functions: Function 1 (LD1), which separated the D60 group from the D20 and non-fighting group, explained 69.1% of the variance, and Function 2 (LD2), which separated the non-fighting group from the D60 and D20 groups, explained 30.9% of the variance (Fig 2B). (**Line 162-165**).

**General concerns about WGCNA:**

**(1)** **I find it highly likely that the three groups differ in the co-expression structure (especially the control group compared to the fighters, but perhaps D20 and D60 as well). I therefore would not combine all the animals into one WGCNA analysis, as the differential expression between groups should drive the correlations identified and lead directly to findings such as close associations between modules and DEGs as reported in lines 218-219 and discussed in 459-470.**

First of all, we noticed that we mistakenly left out the WGCNA description in the Methods in the previous manuscript, which confused the readers. In this revision, we have described the WGCNA in more detail, and we also provided the source code for replication. Please refer to this section and consider whether our description addresses your concerns. If you have questions that remain, please tell us how/where you find it highly likely that the three fighting groups differ in their co-expression structure so that we can answer your question properly.

We should emphasize that the DEG analysis and the WGCNA use different approaches in which the DEG was generated by comparing between the fighting groups (D20 and D60) relative to the baseline (B), whereas the WGCNA was obtained using all the datasets without any comparisons. Here, we used both approaches to analyze the same dataset and had consistent findings across the analyses, which strengthened our conclusions.

**(2) Line 200 states that the associations between the eigengene and fighting groups were computed using correlations. I am not sure based on this phrasing what analyses were conducted, but I don’t understand how correlations could be applied in this context.**

Here, we think that the missing description about the WGCNA in the previous manuscript may have led to your concern. Please see the description about how the WGCNA works in the Methods, which we have newly added in this revision (**Line 657**). If, after reading this section, you have any remaining concerns, please kindly let us know.

**General concern about transcriptomic synchronization:**

**My largest concern with the paper is the transcriptomic synchronization analysis (I adopt the authors’ terminology here, but see my suggestion about this word choice below). The analyses assess whether the transcriptomes of two paired fish have more in common than the transcriptomes of two random fish from unknown behavioral state (so fish that fight have more in common than noise, basically). I’d expect any two fish in the D20 or D60 group – not just those that fought each other – to appear synchronized by this measure.** **If the manuscript wants to assess how similar the transcriptomes of paired fish are, I recommend revising the analysis to compare the paired fish to unpaired fish from that same treatment, omitting the comparison to the non-fighting fish. Figure S4 does suggest this would be a promising approach, but the t-test of the correlation coefficients presented in Figure S4 is not appropriate because the individual pairwise correlations are not independent of one another. Permutation/resampling approaches would offer appropriate alternatives to make general comparisons as to how much paired fish were synchronized as a first step to warrant follow-up characterization of PINS (e.g. permuting which fish are paired then comparing a metric summarizing all correlations of the two transcriptomes, not the correlations in each gene individually). To identify the PINS, I would suggest designing another permutation or resampling test to set a threshold for which genes are PINS rather than relying on a t-test – both correcting for multiple hypotheses and also not requiring the data meet the assumptions of the t test. Because of this fundamental disagreement with the methods used to identify PINS, I have not commented on the broader discussion of the PINS themselves.**

We learned much from your suggestions concerning this analysis and found them very valuable. We greatly appreciate your comments. Accordingly, we re-analyzed our data and re-made (Fig 5A) and revised the interpretation of our results in this version of the manuscript. To assess how similar the transcriptomes of paired fish are, we have revised the analysis to compare the paired fish to unpaired fish from the same treatment (e.g., D20-paired vs. D20-unpaired and D60-paired vs. D60-unpaired) using a permutation test instead of a t-test as in the previous manuscript. Furthermore, we omitted the comparison to the non-fighting fish. We also designed the permutation test to set the threshold for identifying synchronized genes and described this threshold in detail in the Methods. Please have a look (**Line 671-698**).

**If a revised analysis as proposed found** **that transcriptomes for paired fish were more synchronized than fish selected at random, I suggest that the authors consider all possible cause and effect relationships, as the paired fish had very similar behavioral repertoires overall so any** **transcriptomic commonalities could reflect transcriptomic responses to fighting effort or allocation of that effort rather than being related to behavioral synchrony per se (considering that the behaviors were measured quite a bit before the mRNA expression, and the mRNAs would typically influence physiology only after protein synthesis and trafficking).**

In our revised analysis based on your suggestions above, we found that transcriptomes for paired fish were more synchronized than those for fish selected at random from the same group. We thus considered that transcriptomic commonalities reflect transcriptomic responses to fighting effort and have discussed this in the Discussion (see Brain-transcriptomic synchronization reflects cooperative aspect).

**Specific comments:**

**Line 57-58: I don’t see the link between data presented and facial convergence or empathy.**

We have changed the text in this version of the manuscript.

**Before:** This study presents the possibility that animals’ similar behavior under a similar condition triggers to synchronize their waves of transcription between two individuals of a pair of animals in general, providing a hint to explain facial convergence of spouses and empathy in primates on the molecular bases

**After:** This study presents the possibility that animals’ similar behavior under a similar condition triggers to synchronize their waves of transcription between two individuals of a pair of animals in general, providing a hint to support the idea that fighting behavior contain cooperative aspects at the molecular bases. (**Line 66-59**).

**Line 178-180: State method/evidence for this statement**

We have added references for this statement.

**Before:** Noticeably, most of DEGs were up-regulated in both groups and several of these genes have been previously known as neuronal activity markers such as immediate early genes (IEGs), genes related to neural plasticity, neuronal proliferation and/or differentiation, neurotransmitter transport, and epigenetic mechanisms (Fig 3B).

**After:** Noticeably, most of DEGs were up-regulated in both groups and several of these genes have been previously known as neuronal activity markers such as immediate early genes (IEGs), genes related to neural plasticity, neuronal proliferation and/or differentiation, neurotransmitter transport, and epigenetic mechanisms (Fig 3B) (refs. 6, 48). (**Line 187-190**)

**Line 433-440: References needed for these statements**

We have added references for these statements (**Line 377-390**)

**Line 445-447: The conclusion as stated in this sentence oversteps the evidence presented**

We have deleted the text.

**Before:** Altogether, we suggest that long-term fighting activates memory and learning pathways contributing to PINS (61-63) (Fig 6D; Fig S7).

**After:** Entirely deleted.

**Line 544-547: How long does this overdose with MS222 take? The main concern would be the extent to which gene expression might change during the euthanasia.**

The anesthetic time was ~1 min after exposure to MS-222 at a concentration of 600 ppt. We have added this information to the Methods. (**Line 539**).

**Line 582: I wasn’t sure if D50_21 and D50_22 was an error in naming or whether these fish had only 50 minutes of fighting?**

Yes, this was an error in the previous manuscript. We have corrected the names in this revision.

**Line 578-585: Given that the samples had two difference sequencing methods, I recommend adding a sentence that summarizes the extent to which the methods yielded equivalent results and addressing whether the paired vs single end difference led to any biases in the data. Some sort of MDS or PCA plot color coded by sequencing run might also help assuage concerns.**

We have added text based on your suggestions. Given that the samples were analyzed with two different sequencing methods, we first examined whether the paired-end vs. single-end difference led to any biases in the data using a multidimensional scaling (MDS) plot (Fig S7). The MDS plot color-coded by sequencing run revealed that the two methods resulted in a slight bias or in no bias at all. Then, we evaluated the extent to which the methods yielded equivalent results. To minimize the bias, we used only the forward reads for the paired-end sequencing samples and analyzed all data in single-end mode throughout the pipeline. The results obtained were comparable in terms of mapping rates and the number of genes (after normalization of the data by edgeR) (Table S4). They were also consistent with previous studies (ref. 100) and qualified for further downstream analyses. Please see the Methods (**Line 598-607**).

**Figure 2B has D2_31 listed twice.**

We have re-made this figure and corrected this mistake.

**General stylistic suggestions:**

**Although ‘neurotranscriptomic’ and similar terms are used in the literature, I think the term ‘transcriptomic’ is preferable given that** **brain samples include transcripts from many non-neuronal cells that also have dynamic responses to stimuli and may play important functional roles.**

Thank you for your comment. We agree with your suggestion and have changed the term accordingly. In the revised manuscript we have used the term “brain-transcriptomic” instead of “neurotranscriptomic”.

**Also, aside from the major concern about the data analysis supporting the transcriptomic synchronization, a terminology suggestion is that the word synchronization implies coordinated timing. The transcriptomic analyses might reflect coordination, but I would avoid the term synchronization because we don’t have temporal dynamics here.**

Thank you for your suggestion. What we have shown is that at molecular level we demonstrated that brain-transcriptomic synchronization took place at the earlier stage of fighting (at 20 min) and was strengthened after a longer period of interaction (at 60 min), suggesting the presence of the coordinated timing. Importantly, we clearly demonstrated that there is an existence of temporal dynamic structures at the behavioral level across the fight in various fighting pairs.

Although we could not fully illustrate temporal dynamics of brain-transcriptomic changes at present since we collected samples at only three time points, we think it very likely that such temporal dynamics does exist if we could include more samples collected from various fighting time points (e.g. 5 min, 10 min, 20 min, 40 min. 60min etc.,), taking the continuation of fighting and the presence of temporal dynamics at the behavioral level into consideration.

In addition, given that “neural activity synchronization” is widely used in neuroscience, and that correlated brain gene expression may support synchronous neural activity in brains between two interacting partners, we think that it is reasonable to use the term “brain-transcriptomic synchronization”, and we decided to continue to use the term in the revised manuscript.

**Reviewer #3:**

General comments:

**1) Behavioral and physiological synchronization have long been studied by scientists. Although the authors cite a few studies in the Discussion, their treatment of this phenomenon is superficial, given its importance in reproductive/mating behavior, cooperative behavior, aggressive behavior etc. I suggest the topic is best introduced in the Introduction. Further, the recent papers from the labs of Weizhe Hong and Michael Yartsev (which the authors cite later) provide excellent physiological evidence for synchronization. Decades of behavioral evidence as well as these recent physiological findings suggest synchronization at the gene expression levels as a hypothesis.**

We agree that our coverage of previous research on synchronization at the behavioral and physiological level was superficial in the previous manuscript, as you pointed out. Based on your helpful suggestions, we have introduced more detailed information in the Introduction. Please have a look (**Line 105-114**).

**Before:** Did not cover

**After:** Behavioral and physiological synchronization have long been studied by scientists given its importance in reproductive/mating behavior, cooperative behavior, aggressive behavior etc., (33, 34). Some crucial signaling molecules such as serotonin (5-HT) or Arginine-vasotocin (AVT) has been known to modulate the levels of such cooperation at physiological level (35-37). Further, recent studies have provided excellent physiological evidence for such synchronization at the neuronal level (38, 39). Collectively, decades of behavioral evidence as well as these recent physiological findings suggest that synchronization at the gene expression level might also occur.

**2) The authors examined whole-brain transcriptomes. It would be good to provide a rationale for this decision and discuss its limitations. Then, in the Discussion, when pointing at future experiments, consider which brain regions might be good candidates for further analysis.**

We have added this information to the Discussion as you suggested. We stated that none of the most well-known candidate genes for aggressive behavior in zebrafish were identified in our comparison of B vs. D60 (FDR < 0.05 and |log FC| > 2), including, for example, *tphs*, which has key roles in aggression (ref. 28). Therefore, these data prompted us to consider whether aggression-associated genes may have been up- or down-regulated in a specific part of the brain but were not detected in our study of whole-brain gene expression. We also mentioned that studies focusing on specialized parts of the brain known as the “social behavior network”, a collection of brain nuclei that regulate social behaviors across vertebrates, could prove to be more fruitful. Furthermore, we proposed that the telencephalon region could be a good candidate for future analysis because it contains structures that are homologous to the hippocampus and the amygdala in mammals, brain regions known to be associated with aggression in other vertebrates. Particularly, several crucial loci in this region such as the dorsal medial (associated with the fear response), the dorsal lateral (associated with spatial learning), lateral spectrum (associated with anxiety), the pre-optic area (associated with reproduction), the cerebellum (associated with attention) should be analyzed more closely. Please see the Discussion section Whole brain vs. brain regions. (**Line 431-462**).

**3) What is the rationale for selecting the 10,000 most highly expressed genes in the LDA? Why not instead use the 10,000 (or 50%, or similar) most variable genes?**

We found it very helpful for us to use the top 50% most variable genes as you suggested. Thus, we re-made Fig 3B accordingly (please also see Methods).

**Before:** Linear discriminant function analysis (LDA) using the input variables of the top 10,000 highly expressed transcripts from the 25 libraries was used to differentiate the fighting groups (see Methods)

**After:** Linear discriminant function analysis (LDA) using the top 50% most variables of the gene transcripts from the 25 libraries was used to differentiate the fighting groups (see Methods) (**Line 644**)

**4) The asymmetry in gene expression variation between baseline controls and D20 / D60 animals is quite striking (Figure 2C/D). How should we interpret this strong bias towards more highly expressed genes in the fighting animals? How does this result compare to biased transcriptomes described previously in other systems?**

To explain this strong bias in gene up-regulation in the fighting animals, we first clarified that those up-regulated genes in D20 and D60 are immediate early genes (IEGs; e.g., *npas4a*, *cfos*, *nra41*), the expression of which is induced after the first transcriptional wave after neuronal activation (refs 48). These genes orchestrate plasticity at the neuronal, behavioral, and perceptual level. We also mentioned that the most highly expressed gene in both groups is *thoc2*, which is required for efficient export of polyadenylated RNA and spliced mRNA. Our results are consistent with previous studies in sticklebacks (ref. 70), mice (ref. 71), and honeybees (ref. 72) to some extent. In those studies, the isolated DEGs were involved in insulin signaling, oxidative phosphorylation, mitochondrial function, and regulation of metabolic process**es**. Considered together, the highly expressed genes in the D20 and D60 fish suggest a shift in metabolism that prefigures later neuronal plasticity in the brain. (**Line 391-400**).

**Also, consider showing volcano plots for both B vs D20 and B vs D60, and consider volcano plots where the D20 DEGs are projected onto the B vs D60 volcano plots and vice versa.**

We have made volcano plots for the B vs. D20 and B vs. D60 comparisons (Fig 2D) and have shown projections between the D20 and D60 data (Fig 2E) as you suggested. We have described them in detail in the Result section. Please have a look. (**Line 175-181**).

**5) I am not convinced there is good reason to introduce a new acronym “PINS.”** **What the authors are showing is increased covariance in gene expression between combatants compared to baseline controls, especially at the 60 min mark. They interpret this covariance as “synchronization,” which is fine but should be limited to the Discussion. “PINS” seems a little vacuous and excessively speculative.**

Thank you for this comment. We agree that what we showed is increased covariance in gene expression between combatants compared to baseline controls. However, this can only be applied to the case when we take only the DEG analysis into consideration. In addition to the DEG data, however, we demonstrated that the brain-transcriptomic synchronization was also observed by the WGCNA and GoM analyses using the entire dataset without comparing to the baseline controls or making any other comparisons. As we discussed, these different approaches reached the same conclusion. Additionally, in Fig 6D and Fig S4C we emphasized that although those synchronized genes were found in all five fighting pairs, their synchrony levels differed in a pair-specific manner. Collectively, we think that it is worth introducing the term PINS; however, we agree to limit this term to the Discussion and instead of introducing it in the Results. Notably, in this revision we replaced the term “**N**eurotranscriptomic” with the term “**B**rain-transcriptomic” throughout the manuscript, given that transcriptomes from non-neuronal cells might also contribute to the synchronization. Thus, in this revision “PINS” was replaced by “PIBS”.

**6) Do the observed (synchronized)** **gene expression patterns give insight into the neuromolecular mechanisms underlying sequential assessment, as laid out by Enquist & Leimar sand many others since then?**

As we discussed in the section “Brain-transcriptomic synchronization reflects cooperative aspect” of the Discussion, we do think that gene expression patterns give insight into the neuromolecular mechanisms underlying sequential assessment to seek cooperation that benefits both interacting opponents. Please see the Discussion. (**Line 463-486**).

**7) The survey of DEG promoters in search of overrepresented transcription factor binding sites is superficial and distracting. It does not add much to the study, so I suggest either to remove it entirely or to move it to supplemental materials.**

We agree that the promoter analysis distracted from the focus of our study and have removed it entirely in this revision, as you suggested.

Specific comments:

**8) What was the rationale for choosing the 20 min and 60 min time points, respectively?**

We noticed that we did not mention the rationale for choosing the 20-min and 60-min time points in the previous manuscript. Thus, we have added the rationale to this revision. Please see the Introduction (**Line 121- 124**).

**Before:** Did not cover

**After:** While the 20-min fight allows us to capture early response genes (IEGs) – known as genomic markers for brain activity and marked new experience for fish, the 60-min fight permits us to evaluate neurogenomic responses of full range of volitional behavior responding to its opponent over the course of the encounter.

**9) Figure 3C: This heatmap is fine, though the legend is illegible. I would like to see hierarchical clustering of all samples for, say, the top 50% most variable genes. How clearly are the three groups (B, D20, D60) separated? Do paired individuals cluster together? I believe the heatmap in Figure 5B attempts this (though why is it part of Figure 5?), but has way too many uninformative (i.e., barely variable) genes and the dendrogram has not been bootstrapped. Like all other clustered heatmaps in this study, dendrogram should have bootstrap values (consider using R package pvclust).**

Fig 3C and Fig 5B were designed to serve different purposes. In Fig 3C, we focused on demonstrating expression differences between the fighting groups and showing only those genes that showed significant regulation (FDR < 0.05 and |log FC| > 2). In contrast, in Fig 5B we focused on expression differences between individuals within and between pairs using all genes.

In this revision, we decided to retain Fig 3C to which we added bootstrap and confidence value for the cluster node). We agree that Fig 5B had too many uninformative (i.e., barely variable) genes and that the dendrogram had not been bootstrapped. Thus, we re-made the figure based on your suggestions by using the top 10% most variable genes (2,330 genes) and adding the dendrogram with bootstrap values. Please have a look.

It should be emphasized that clustering of gene expression changes between two opponents within a fighting pair in the D60 group was observed even when the entire gene set (23,306 genes) was used, which included several barely variable genes, supporting our claim for the presence of pair-specific individualization of brain-transcriptomic synchronization. We have mentioned this in the revised version of the manuscript and provided the heatmap in the supplemental figure. (**Line 229-233**).

**10) What is the co-variance structure between module eigengenes and various behavioral metrics?**

Thank you for this comment. Again, the WGCNA description in the Methods may clarify this point. We have also included the behavioral metrics in this revision (see Table S15).

**11) The GoM analysis is very cool and convincing! Are there any test statistics that would allow us to estimate the strength of the association? Is there a way** **to quantify the strength of association between GoM and behavioral synchronization of any given pair?**

The GoM analysis appears to be valuable for visualizing RNA-seq data, as we mentioned in the Discussion. We believe that there should be a way to quantify the strength of association between GoM and behavioral synchronization for any given pair, as you mentioned. However, our behavioral analysis in this study was not comprehensive enough to allow such an analysis. Several other sets of behaviors that could also require such cooperation, for example, tail-beating, body-orientation, body-coloration, were not examined due to their complexity. Therefore, at present, we cannot quantify the association between GoM and behavioral synchronization for any given pair. We have discussed this limitation in the revision, while noting that this is a point we expect to address in a future study.

**12) While much talked about, the “mirror neuron” concept is quite contentious among systems neuroscientists, with good reason to think that they are an artefact. I strongly suggest removing this discussion.**

We agree that the mirror neuron concept is contentious and have removed this term from this version of the manuscript.

We would like to add that several lines of evidence have recently emerged to support the coupling of interbrain neural activity between interacting partners. Thus, in this revision we included these points in the section “Brain-transcriptomic synchronization reflects cooperative fighting aspect” in the Discussion. We aimed to add more information to support the idea that brain-transcriptomic synchronization could provide insights into the neuromolecular mechanisms underlying sequential assessment.

**Before:** PINS Linked to Mirror Neurons and Empathy

**After:** Brain-transcriptomic synchronization reflects cooperative fighting aspect.
