# Supplementary material for: Behavioral and brain- transcriptomic synchronization between the two opponents of a fighting pair of the fish Betta splendens
Source: PLoS Genet. 2020 Jun 17;16(6):e1008831. doi: 10.1371/journal.pgen.1008831 (PMC7299326; doi:10.1371/journal.pgen.1008831)
Supplement: Attachment — Submitted filename: 200428_reviewer_ responses.docx [file pgen.1008831.s025.docx]

**Dear** **Drs. Alison Bell (Guest Editor) and Gregory Barsh (Editor-in-Chief):**

We greatly appreciate the constructive comments and suggestions made by you and the two anonymous reviewers, which were very helpful in improving our manuscript. We have tried our best to respond to these comments and have revised our manuscript accordingly.

One of the improvements in this revised version is that we added the description of the principal component analysis (PCA) result (please see the Fig S4E in the Supplementary figure file), which was performed by using the same dataset (11,653 genes) as that for the linear discriminant function analysis (LDA) in the previous manuscript (Fig 2B). Moreover, we transferred the original Fig S8 to the main text as Fig 6D (left) considering that it is more illustrative for readers to understand what is pair-specific individualization of brain-transcriptomic synchronization. Finally, we added information and modified the interpretation of the rationale for using whole brain given that it seems to require more explanation.

Regarding a cover image, we attached two photos here, which are very illustrative for fighting behavior of this fish. They are commercially available. If the office can agree with using one of these photos, we are going to buy it. Having said that, there will be no license problem with it.

Below are our changes and point-by-point responses to the comments:

**Editors**

1. **Line 42: please replace “higher” with “vertebrate”**

We have changed accordingly, please see line 45

2. **Line 72: females also fight. Please remove “male” and change “conspecific” to “conspecifics”**

We have changed accordingly, please see line 74

3. **Line 109: remove “excellent”**

We have changed accordingly, please see line 112

4. **Line 173: change “discover” to “discovery”**

We have changed accordingly, please see line 180

5. **Line 428: remove “genomics”; integrative genomics typically refers to integrating different types of “omics data, e.g. RNASeq, DNASeq, ATAC-Seq, etc.**

We have changed accordingly, please see line 440

**Reviewer # 3**

**1. I suggest the authors provide a couple of clear hypotheses or predictions before they lunch into the Results. This would be very much help with the overall framing of the study.**

Thank you for your suggestion. We agree that providing a couple of clear hypotheses or predictions could aid the better understanding of the overall framing of this study. Thus, we have newly added two specific questions in this revision (please see page 5, lines 116-119), while retaining the previous general prediction.

**Before:** not mention

**After:** We ask the following questions **1**) what is the relationship between brain-transcriptomic activity in two individuals that are engaged in social interactions? **2**) how does this relationship change across different timescales to facilitate social interactions?

**2. Because the linear discriminant analysis (LDA) is supervised,** **it might be useful to also use principal component analysis (PCA) on this dataset, especially given that the authors** **did not have clear expectation on the clustering.**

Both LDA and PCA analyses are commonly used as dimensionality reduction technique for pattern classification. As you suggested that LDA is a supervised method, and that it might be also useful to use principal component analysis (PCA), which is unsupervised. We appreciate this suggestion and make a change accordingly.

In this revision, a PCA has been done on the same dataset as that applied for the LDA (11,653 genes), and the LDA and PCA plots were shown in the Fig 2B (in the manuscript) and Fig S4E (in the Supplementary figure file), respectively. Intuitively, our result showed that the LDA is better illustrative than the PCA in terms of classification of the fighting groups. This makes sense given that the goal of the LDA analysis is to find the axes that maximize the separation between multiple classes, whereas the goal of the PCA analysis is to find the axes that maximize the variance in a dataset since it ignores class labels.

Importantly, we found that the paired fish in the D60 group were closely clustered together by the PCA, which we described in this revised manuscript (please see lines 168-170). Therefore, we have added the data and the methodology of PCA analysis to the sections for Supplementary (Fig S4E) and Method, respectively.

**Before**: did not mention

**After**: To differentiate transcriptomic responses among the three groups (B, D20, and D60), a principal component analysis (PCA) and a linear discriminant analysis (LDA) were done on the same data set of the top 50% most variable gene transcripts (11,653 genes sorted by standard deviation) from the entire gene set after undergoing DEG analysis in edgeR (n = 23,306 genes). Both the PCA and LDA analyses were implemented using the "MASS" package in R (Fig S4E; source code, LDA). Please see lines 666 - 671.

**3. The rationale for using whole brain is improved, though it seems largely based on hindsight, given the results of this study. I still think the authors made an appropriate decision here, but they can justify it better by pointing at previous studies that did whole brain gene expression profiling in the context of aggression or social status.**

We highly appreciate your suggestion. We have newly interpreted our rationale for using whole brain in this manuscript accordingly.

**Before**: Please see lines 432 - 462

**After**: Basically, we first provide a rationale for using whole brain in which it is an organ where social experience is integrated with physiological changes to carve the phenotypic plasticity. Next, we discuss its limitations reasoning that brain tissue is a complex mixture of neuronal cell types, each of which has different biological signals, and that some of meaningful signals may be undetected because of their relative low abundance. Then, we suggest that this limitation can be overcome by obtaining RNA from smaller area of the brain using microdissection or single cell sequencing method. Finally, we suggest that the telencephalon region and several loci might be good candidates for further analysis. Please see lines 449 - 463.

**4. On lines 391ff. the authors now address the asymmetry in DEGs they discovered, suggesting “the possibility of a shift in metabolism that prefigures later neuronal plasticity in the brain.” This is an intriguing idea, though I wonder how generalizable it is (see, e.g., Cummings et al., ProcRSoc B 275:393, 2008, where fewer genes were highly expressed in the ostensibly more “salient” stimulus situation). Is it possible that the asymmetry in DEGs between baseline and fighting groups is a molecular manifestation of increased inhibitory control of gene expression on aggression (so as to avoid an over-escalation), as suggested by Wong & Hofmann, Encyclopedia of Life Sciences, John Wiley & Sons, Ltd: Chichester. DOI: 10.1002/9780470015902.a0022554, 2010)? Also, how could any of these ideas be tested? This is obviously outside of the scope of this study, but I wanted to share these thoughts with the authors.**

We agreed with your suggestion and we have added the suggested information and cited the reference in this manuscript accordingly.

**Before:** not mention

**After:** it is also possible that the asymmetry in DEGs between baseline and fighting groups could be a molecular manifestation of increased inhibitory control of gene expression on aggression so as to avoid an over-escalation [73]. Please see lines 410 - 412.

5. **Consider including Fig S8 in the main paper**

We agreed with your suggestion and made a change accordingly. We turned Fig S8 into the main Fig 6D.

**Before**: please see supplementary Fig S8 in the supplementary figure file

**After**: please see Fig 6D (left) in this revised manuscript
